# Metabolism and development – integration of micro computed tomography data and metabolite profiling reveals metabolic reprogramming from floral initiation to silique development

**DOI:** 10.1111/nph.12631

**Published:** 2013-12-18

**Authors:** Anke Bellaire, Till Ischebeck, Yannick Staedler, Isabell Weinhaeuser, Andrea Mair, Sriram Parameswaran, Toshiro Ito, Jürg Schönenberger, Wolfram Weckwerth

**Affiliations:** 1 Department of Structural and Functional Botany, Faculty of Life Sciences, University of ViennaRennweg 14, Vienna, Austria; 2 Department of Ecogenomics and Systems Biology, Faculty of Life Sciences, University of ViennaAlthanstrasse 14, Vienna, Austria; 3 Temasek Life Sciences Laboratory, National University of SingaporeSingapore, Singapore

**Keywords:** *Arabidopsis thaliana*, basic leucine zipper (bZIP) transcription factors, flower development, metabolomics, morphometry, multivariate statistics, ontogenetic trajectory, sucrose sensing

## Abstract

- The interrelationship of morphogenesis and metabolism is a poorly studied phenomenon. The main paradigm is that development is controlled by gene expression. The aim of the present study was to correlate metabolism to early and late stages of flower and fruit development in order to provide the basis for the identification of metabolic adjustment and limitations.
- A highly detailed picture of morphogenesis is achieved using nondestructive micro computed tomography. This technique was used to quantify morphometric parameters of early and late flower development in an *Arabidopsis thaliana* mutant with synchronized flower initiation. The synchronized flower phenotype made it possible to sample enough early floral tissue otherwise not accessible for metabolomic analysis.
- The integration of metabolomic and morphometric data enabled the correlation of metabolic signatures with the process of flower morphogenesis. These signatures changed significantly during development, indicating a pronounced metabolic reprogramming in the tissue. Distinct sets of metabolites involved in these processes were identified and were linked to the findings of previous gene expression studies of flower development. High correlations with basic leucine zipper (bZIP) transcription factors and nitrogen metabolism genes involved in the control of metabolic carbon : nitrogen partitioning were revealed.
- Based on these observations a model for metabolic adjustment during flower development is proposed.

## Introduction

Angiosperms are the largest group of higher plants, with 300 000–400 000 estimated species (The Angiosperm Phylogeny Group, 2009). Flower and fruit development in these plants are the most critical steps of generative reproduction. The understanding of developmental processes involved in flower formation and reproduction is particularly important, as the major part of our nutrition directly or indirectly relies on angiosperms (Dwivedi *et al.*, 2008; Weckwerth, 2011).

Flower and fruit development are usually characterized by the following major structural changes: floral initiation, floral organ initiation, floral morphogenesis, floral differentiation and growth, anthesis, fruit differentiation and growth leading finally to fruit maturity (Endress, 1994).

The morphology of flower development of *Arabidopsis thaliana*, a species of the Brassicaceae family and a model species in plant sciences, has been analysed by Smyth *et al*., who defined 20 stages from the appearance of the first floral meristem to the dispersal of seeds (Müller, 1961; Smyth *et al.*, 1990). These stages are summarized in Table 1.

**Table 1:** Morphometric data for *Arabidopsis thaliana pAP1::AP1-GR ap1-1 cal-5* flower development and silique formation; see Materials and Methods for more details on the plant line

Days after initiation of flower development	Stage according to Smyth *et al*. (1990)	Number of flowers (of *n* samples)	Flower length (μm) (cv)	Gynoecium width, septum (μm) (cv)	Gynoecium width, dorsiventral (μm) (cv)	Gynoecium length (μm) (cv)	Anther width, long stamen (μm) (cv)	Anther length, long stamen (μm) (cv)	Filament length, long stamen (μm) (cv)	Anther width, short stamen (μm) (cv)	Anther length, short stamen (μm) (cv)	Filament length, short stamen (μm) (cv)
0	1	14 (2)	35 (22%)	0	0	0	0	0	0	0	0	0
2	2	15 (2)	48 (10%)	0	0	0	0	0	0	0	0	0
4	3	5 (1)	44 (14%)	0	0	0	0	0	0	0	0	0
6	4; 5; 6	5 (1)	98 (10%)	52 (12%)	65 (10%)	55 (15%)	48 (21%)	49 (24%)	0	0	0	0
8	7; 8	5 (1)	367 (26%)	87 (14%)	107 (11%)	314 (28%)	149 (16%)	152 (21%)	156 (28%)	118 (9%)	134 (15%)	135 (5%)
10	9	6 (1)	386 (21%)	83 (17%)	99 (15%)	342 (25%)	147 (21%)	172 (14%)	187 (19%)	139 (7%)	142 (17%)	140 (15%)
12	10	5 (1)	654 (13%)	141 (6%)	148 (5%)	567 (11%)	223 (3%)	212 (17%)	250 (11%)	196 (8%)	240 (4%)	207 (7%)
14	11	5 (5)	1055 (13%)	250 (19%)	251 (19%)	888 (6%)	311 (6%)	315 (6%)	384 (7%)	297 (2%)	312 (3%)	334 (4%)
16	12	5 (5)	1306 (4%)	238 (11%)	252 (13%)	1187 (5%)	319 (5%)	330 (8%)	443 (13%)	304 (2%)	319 (4%)	356 (2%)
18	13	5 (5)	1975 (15%)	295 (5%)	329 (6%)	1831 (15%)	322 (10%)	359 (11%)	1415 (32%)	316 (2%)	342 (3%)	1097 (8%)
20	15	5 (5)	3070 (12%)	317 (3%)	377 (7%)	2774 (17%)	269 (3%)	344 (7%)	1682 (13%)	278 (3%)	334 (7%)	1439 (1%)
22	16	5 (5)	7640 (15%)	402 (9%)	491 (8%)	6903 (14%)	0	0	0	0	0	0
24	17	5 (5)	7557 (13%)	469 (23%)	553 (14%)	7310 (14%)	0	0	0	0	0	0
27	17	5 (5)	8167 (14%)	537 (13%)	716 (17%)	7989 (15%)	0	0	0	0	0	0

All the data were obtained based on micro computed tomography (micro-CT) scans. cv, coefficient of variation, calculated as (standard deviation: mean) × 100.

The genetic control of floral development in *A. thaliana* has also been investigated (Bowman *et al.*, 1989, 2012; Coen & Meyerowitz, 1991; Coen, 1992; Meyerowitz, 1994; Wellmer *et al.*, 2006).

Yet, a detailed metabolic profile of all major steps of flower development, from floral organ initiation to fruit development, has not been reported to date. This is especially relevant because it has been demonstrated that plant tissues are able to sense metabolic states in order to initiate or control specific developmental and growth processes (Muller *et al.*, 1999; Rolland *et al.*, 2006; Sakakibara *et al.*, 2006; Hanson *et al.*, 2008; Smeekens *et al.*, 2010; Ma *et al.*, 2011; Wahl *et al.*, 2013). These sensing mechanisms involve complex signal cascades and transcription factor regulation based on sugar and energy levels within tissues.

**Fig 1:**
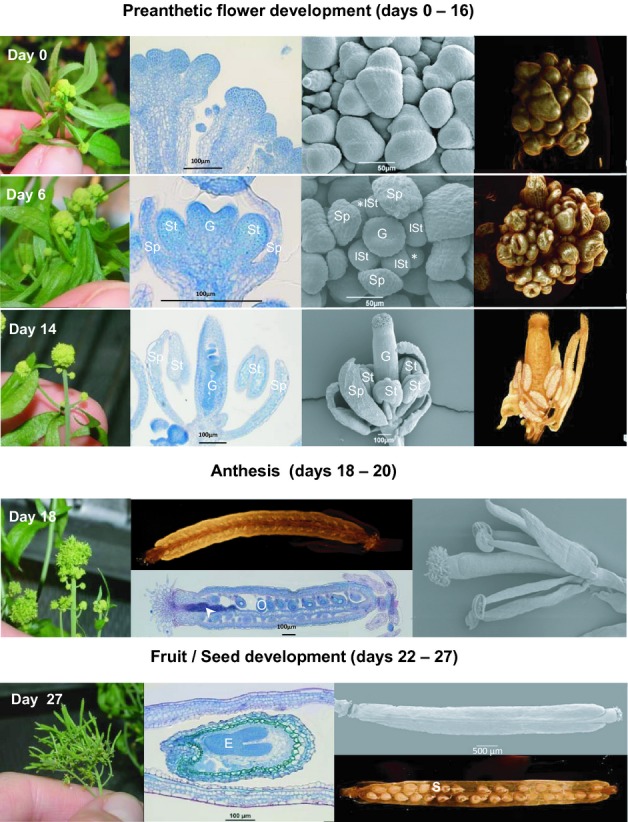
Flower development of *Arabidopsis thalianapAP1::AP1-GR ap1-1 cal-5* plants was monitored after dexamethasone induction (day 0) using the following methods (left to right): photography, light microscopy of longitudinal sections stained with ruthenium red and toluidin blue, scanning electron microscopy and micro computed tomography (micro-CT). Sp, sepal; lSt, long stamen; *, short stamen; G, gynoecium; the arrowhead indicates pollen tube transmitting tissue; O, ovule; S, seed; E, embryo.

The aim of the present study was to correlate metabolism with morphological stages from flower initiation to fruit formation. To accomplish this goal, various stages of flower development of *A. thaliana* were investigated with scanning electron microscopy (SEM), light microscopy (LM) and X-ray micro computed tomography (micro-CT) (Fig.1). Micro-CT is typically used in biomedical analysis but is increasingly applied to fundamental research in organismal biology and plant sciences (Tappero *et al.*, 2007; Kaminuma *et al.*, 2008; Dhondt *et al.*, 2010; Brodersen *et al.*, 2011; Carey *et al.*, 2011; Blonder *et al.*, 2012; Gregg & Butcher, 2012; Gamisch *et al.*, 2013; Pajor *et al.*, 2013; Staedler *et al.*, 2013). Because of its nondestructive character and its ability to reveal the quasi-native 3D structure of samples, micro-CT is an ideal technology to bridge the gap between morphological and molecular studies. Complementarily to these structural measurements, metabolites present in the same developmental stages of *A. thaliana* flowers and siliques were analysed by gas chromatography coupled to mass spectrometry (GC-MS). In order to obtain enough material of the earliest developmental stages, a mutant line with synchronized inflorescences was used: the floral development of the *pAP1::AP1-GR ap1-1 cal-5* mutant (*APETALA1* (*AP1*) endogenous promoter-driven *AP1* gene with a carboxy-terminal fusion to the steroid-binding domain of the rat glucocorticoid receptor (*GR*) in the double mutant background of *ap1-1* and *cauliflower-5*; see Materials and Methods for more details on the plant line) is arrested at a very early stage, until induced with dexamethasone (Wellmer *et al.*, 2006). This mutant displays normal flower development with the exception of occasionally reduced or missing petals. To a minor degree, this also applies to the androecium, where single stamens can be missing sometimes. However, this does not influence the development of the remaining organs, and the flowers produce fertile seeds. This allowed us to harvest enough tissue for each developmental stage, and to establish a protocol for metabolite analysis of very early stages of development, beginning with floral initiation. Multivariate statistical analysis was used to visualize the combined morphometric and metabolic trajectory of flower development in *A. thaliana*. This combined analysis of covariance patterns – demonstrated recently as a general data integration strategy (Weckwerth *et al.*, 2004; Morgenthal *et al.*, 2005; Wienkoop *et al.*, 2008; Valledor *et al.*, 2013) – allowed the identification of metabolic signatures, which are strongly associated with specific stages of flower and silique development. These metabolic signatures were further compared with gene expression analysis of very early stages of flower development from previous studies (Wellmer *et al.*, 2006) revealing a potential metabolic sensing mechanism and adjustment of the carbon (C) : nitrogen (N) ratio in plant flower tissue based on the basic leucine zipper transcription factor 11 (bZIP11) and genes of the nitrogen assimilation pathways, for example, glutamine synthetase and glutamate dehydrogenase.

## Materials and Methods

### *Arabidopsis thaliana* mutant with synchronized flower development

The *Arabidopsis thaliana* (L.) Heynh. mutant *pAP1::AP1-GR ap1-1 cal-5* was constructed as follows: the genomic sequence of *A. thaliana AP1* was amplified from a *bacterial artificial chromosome (BAC)* clone template *F4N2*, corresponding to clone *AC008262*,*Arabidopsis thaliana chromosome one (AtChr1)*, to generate a *Kpn*I site and an *Apa*I site at the 5′ and 3′ ends, respectively, resulting in a 6118-bp fragment. The fragment was first cloned into vector pCR2.1-TOPO® (Invitrogen, Carlsbad, CA, USA) and then transferred to vector *pTI0280* (at *Asp718/Apa*I sites) which harbours *35S::GR*, and eventually cloned into the *Not*I site of vector *pART*. The construct was then transformed into the *ap1-1 cal-5* double mutant via *Agrobacterium tumefaciens*-mediated transformation. Successful transformants were screened with kanamycin. Like in *ap1-1 cal-5* plants, in *pAP1::AP1-GR ap1-1 cal-5* flower development is arrested in a very early stage (stage 1; Table 1). It can be rescued by spraying the arrested inflorescences with dexamethasone. This leads to the activation of the AP1-GR protein and to synchronized flower development, as all flowers continue their development simultaneously (for further details see also Ferrandiz *et al.*, 2000; Wellmer *et al.*, 2006). The mutant is partially affected in corolla and androecium development (petals are not fully developed or missing, and short stamens are sometimes missing). Other aspects of development and the production of fertile seeds are not affected (see Fig.1, Table 1).

### Plant growth and harvest

*Arabidopsis thaliana pAP1::AP1-GR ap1-1 cal-5* individuals were grown in a phytotron for 6 wk under short-day conditions (8 h light: 16 h darkness), followed by 2 wk under long-day conditions (16 h : 8 h, light : dark) (night temperature 20°C; day temperature 22°C). Synchronized flower development was induced by spraying the plants once with the synthetic hormone dexamethasone, which led to the activation of *AP-1-GR* and thereby to the start of floral development. The day on which the plants were sprayed was also the first day of harvest (day 0) (see Table 1).

Subsequently, the inflorescences of the main apices were harvested every 2 d, providing 14 different stages of development. Fifty-six samples of inflorescences were preserved in FAA (formaldehyde, acetic acid and alcohol; 70% ethanol : acetic acid : 40% formaldehyde 90 : 5 : 5, v/v/v) for morphological and morphometric analysis. Seventy samples with a fresh weight of 20 mg were fixed in liquid N for metabolomic analysis.

### Light microscopy (LM)

For microtome thin sectioning, the FAA-fixed specimens were dehydrated in an ethanol series (85 then 96%), evacuated and embedded in 2-hydroxyethyl methacrylate (Kulzer's Technovit 7100; Heraeus Kulzer, Wehrheim, Germany). Sectioning was performed with a rotary microtome (Microm HM 355, Thermo Scientific, Walldorf, Germany) at 5 μm followed by successive staining with ruthenium red and toluidine blue. More detailed descriptions of embedding, sectioning, and staining methods are given in Igersheim (1993) and Igersheim & Cichocki (1996). Sections were mounted permanently in Enthellan (Merck, Darmstadt, Germany).

Microscopic observations were made with a light microscope with ×40/0.85 objectives (System Microscope BX 50; Olympus, London, UK) using bright field. The images were captured with a digital camera (DS-Fi1; Nikon, Tokyo, Japan) combined with a PC-based microscope camera control unit (DS-U2; Nikon) and seamlessly integrated with the imaging software suit (NIS-Elements D3.2; Nikon). For further processing, the images were transferred to Photoshop (Adobe Systems Inc., San Jose, CA, USA).

### Scanning electron microscopy (SEM)

The FAA-fixed material was washed with 70% ethanol and dehydrated in an ethanol series (85 then 96%) and acetone before CO_2_ critical point drying using an Autosamdri-815 (Tousimis, Rockville, MD, USA). The dried samples were mounted on aluminium stubs and coated with gold using a sputter coater (SCD 050; Denton Vacuum LLC, Moorestown, NJ, USA). Images were obtained in a scanning electron microscope (JSM-6390; JEOL, Peabody, MA, USA). For capturing the images, the software sem controle user interface version 8.24 (JEOL, Peabody, MA, USA) was used.

Samples of younger stages (days 0–12) were mounted as primary inflorescences, whereas older stages (days 14–27) were mounted as single flowers and siliques, respectively.

### X-ray micro computed tomography (micro-CT) and morphometric analysis

For X-ray micro-CT, all samples were treated with a solution of 1% (w/v) phosphotungstic acid in FAA for at least 1 wk, changing the solution every second day following the protocols given in Staedler *et al*. (2013). The inflorescences of the first seven samples (days 0–12) were critical point dried and sputter-coated as described in the previous section and glued onto an aluminium holder with two-component epoxy glue. Flowers and siliques harvested from day 14 to day 27 were treated individually, and tightly fixed in an upright position in a pipette tip immersed in solution of 1% (w/v) phosphotungstic acid in FAA.

**Fig 2:**
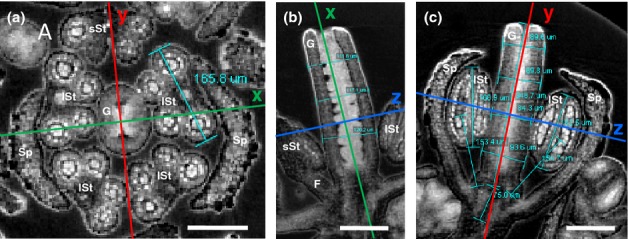
Morphometric parameters of *Arabidopsis thaliana pAP1::AP1-GR ap1-1 cal-5* flower development at day 8 after flower initiation were measured via virtual sectioning of 3D micro computed tomography (micro-CT) images. (a) View onto a *z*-plane cross-section of the gynoecium and anthers. Measured parameters: gynoecium width (septum), gynoecium width (dorsiventral), and anther width. (Note that gynoecium parameters are not shown, and the second short stamen is missing in this flower.) (b) View onto a *y*-plane longitudinal section of the gynoecium. Measured parameter: gynoecium width (dorsiventral). (c) View onto an *x*-plane longitudinal section of the flower. Measured parameters: flower length (sum of gynoecium length and floral base); gynoecium width (septum); gynoecium length; anther length; filament length. *x*,*y* and *z* indicate the position of the corresponding planes. Bars, 100 μm. G, gynoecium; lSt, long stamen; sSt, short stamen; F, filament; Sp, sepal.

The samples were imaged at 0.4–3.7 μm voxel size with an X-ray tomography system (MicroXCT-200; XRadia Inc., Pleasanton, CA, USA). This system uses a 90-kV microfocus X-ray source (L9421-02; Hamamatsu, Hamamatsu City, Japan), a cooled 2 k 2 k CCD camera, and switchable scintillator objective lens units. Scanning settings are summarized in Supporting Information Table S1. The XMReconstructor 8.1.6599 software (XRadia) was used to perform the 3D reconstruction from the scanning data. For samples that were scanned in several steps, the XMController 8.1.6599 software (XRadia) was used to stitch together the resulting scan data. The TMX3D Viewer software (XRadia) was used to perform the morphological and morphometric analyses. In order to carry out exact measurements of the ten developmental parameters (see Table 1), 2D pictures were generated bringing the individual parameters into focus (see also Fig.2 and the Results section Morphometric analysis of flower development using micro-CT and multivariate statistics).

For each of the 14 d of sampling, five to 14 replicates were analysed in this way. For details and statistics, see Table 1.

### Metabolomics using gas chromatography coupled to mass spectrometry (GC-MS)

Metabolomic analyses were performed according to Weckwerth *et al*. (2004). For homogenization, the samples were stored in liquid nitrogen in 2-ml Eppendorf tubes and three metal beads (diameter 1.5 mm) were added. The samples were homogenized in a ball mixer mill (MM40; Retsch GmbH, Haan, Germany) with a maximal speed of 30 movements s^−1^ for 3 min.

Samples were then extracted with 500 μl of a cooled extraction solution of methanol : chloroform : water (2.5 : 2 : 1, v/v/v), and 5 μl of the internal standard ^13^C-sorbitol (1 g l^−1^) was added. The extracts were vortexed, incubated for 8 min on ice and then centrifuged (Heraeus Fresco 21 centrifuge; Heraeus, Newport Pagnell, UK) for 4 min at 20 000 ***g*** at 4°C. The supernatant was then removed and transferred to an Eppendorf tube (2 ml). Water (250 μl) was added, and the solution was centrifuged for phase separation for 2 min at 20 000 ***g*** at 4°C. The upper polar methanol to water phase was transferred to a new Eppendorf tube (1.5 ml) and stored at −20°C. Aliquots of the polar phase containing 2 mg of material were dried in a Speed Vac (Thermo Scientific, Vienna, Austria). Five μl of solution (8 mg l^−1^ methoxyamine hydrochloride in 200 μl of pyridine) was added to the dry polar phase and then shaken on a thermo mixer (Eppendorf AG, Hamburg, Germany) for 90 min at 30°C. The samples were then silylated with 20 μl of MSTFA (Sigma-Aldrich, Vienna, Austria), again mixed on the thermo mixer for 30 min at 37°C and subsequently analysed by GC-MS (Pegasus® 4D GCxGC-TOFMS; Leco, Mönchengladbach, Germany). One μl of the sample was injected at 230°C onto an HP 5-ms capillary column (5%-phenyl)-methylpolysiloxane; 30 m × 0.25 mm ID; standard film thickness of 25 μm), with a 25 split or 10 split mode. Helium was the carrier gas with a flow velocity of 1 ml min^−1^. The temperature gradient started at 70°C for 1 min and increased by 9°C min^−1^, with a final temperature of 350°C maintained for 3 min. The mass-to-charge ratio range was set to 40–600 and the scan rate to 20 s^−1^. Data analysis was performed with Chroma TOF software (Leco). The software compares the mass spectra of the sample with a user-defined spectra library and a retention index thereby delivering peak areas per metabolite. The peak areas were normalized by the internal standard and the fresh weight of the sample.

### Multivariate statistical and bioinformatic data analysis

Data transformation, alignment and integration as well as principal components analysis (PCA), k-means and bi-clustering were performed with the statistical toolbox covain (Sun & Weckwerth, 2012, 2013; Doerfler *et al.*, 2013). The software, a user manual and parameter settings can be downloaded from http://www.univie.ac.at/mosys/software.html. Detailed protocols are also described in Sun & Weckwerth (2013). Further applications of covain can be found in Egelhofer *et al*. (2013), Hoehenwarter *et al*. (2013), Mari *et al*. (2013), Naegele & Weckwerth (2013), Napolitano *et al*. (2013), Obermeyer *et al*. (2013) and Valledor *et al*. (2013). The bi-clustering function implemented in covain uses average linkage of Euclidean distance between groups as the metric.

## Results

### Morphological analysis of *A. thaliana* flower development

Flowers from a total of 14 time-points comprising developmental stages ranging from floral initiation to fruit formation (see Table 1) were harvested for morphological analysis by photography, LM, SEM and micro-CT (see Fig.1). In the *A. thaliana pAP1::AP1-GR ap1-1 cal-5* mutant that was used for analysis, natural flower development is arrested at stage 1 according to Smyth *et al*. (1990) and Müller (1961) (see ‘day 0’ in Fig.1 and Table 1). At this stage, the primordial floral apex is differentiated. Application of dexamethasone initiates the subsequent steps of floral development. A detailed comparison of the developmental phases of the mutant with the wild-type can be found in Table 1. Each developmental stage of the mutant was assigned to a corresponding stage of *A. thaliana* wild-type flowers described by Smyth *et al*. (1990) and Müller (1961), based on a comparison of LM, SEM and micro-CT data.

Three major phases of flower/fruit development can be identified. During the first phase of flower development (days 0–16; preanthetic), all floral organs are established in the usual order of sepals, petals, stamens, and carpels. The four sepal primordia arise and gradually cover the young inner organs (Fig.1; day 6). The petal primordia grow very slowly or – more commonly – are arrested in their development in the mutant, while the stamen primordia grow relatively fast. The anthers of the four long stamens develop first (Fig.1; day 6). Around days 18–20, the filaments of the four long stamens elongate considerably more than those of the two short ones (Table 1). Pollen tetrads are visible between days 8 and 10 (Fig. S1A). The two-carpellate gynoecium is initiated last, but is soon overtopping the stamens (Fig.1; day 14). Initially, the gynoecium is open at its distal end (day 6 to day 12), and the postgenital closure of the carpels is established *c*. day 14. Finally, a short style with a tapering apex is formed. This is also the time when the ovules are almost mature and the stigmatic papillae develop (day 16).

The second phase of flower development, anthesis, occurs at *c*. day 18. During this transition phase, the stigmatic papillae become fully developed and receptive. The pollen tube transmitting tissue within the short style (stained purple-red in the LM picture in Fig.1; day 18) is ready for guiding the pollen tubes to the ovules. The latter are located on parietal placentae in the two locules, which are divided by a false septum (Fig. S1C–F). During this developmental stage, the filaments reach their final length and bear the dehiscing anthers that release their fully developed pollen grains (Fig. S1C,D).

After pollination and fertilization, the flower passes into a third phase which corresponds to fruit and seed development. The perianth and androecium wither, and eventually fall off on day 22. The siliques enlarge rapidly from day 20 to day 22, but more slowly from day 22 to day 27 (see Table 1 and next section). The developing dicotyledonous embryo can be seen soon after day 20. An embryo of the late heart stage is shown in the LM picture in Fig.1 (day 27). The very small cells of the embryo stain intensively with toluidine blue, which indicates that the meristematic tissue is highly active.

### Morphometric analysis of flower development using micro-CT and multivariate statistics

To obtain a spatial impression and direct access to the morphometric parameters of the flower, a micro-CT system was used to produce 3D models of different developmental stages (Fig.1).The reconstructed 3D models of the flowers allow for the dissection of the organs into 2D virtual sections (Fig.2). For morphometric analysis, ten characteristic parameters which displayed significant change throughout flower development were identified: (1) flower length; (2) gynoecium length; (3) gynoecium width in the plane of the septum; (4) gynoecium width in the dorsiventral plane of the carpels; (5) anther length; (6) anther width; and (7) filament length of long and short stamens, respectively. These parameters were measured in the 2D virtual sections (Fig.2).

Flower length was the only parameter that could be measured throughout all developmental stages. In early stages, flower length is defined as the length of the floral apex, and in older stages as the distance between the gynoecium tip and the lowermost point of sepal attachment along the floral axis. Thus, tomographic longitudinal sections in the septal plane of the gynoecium, revealing the level of attachment of the two lower sepals (adaxial and abaxial), were chosen for all measurements of flower length (Fig.2c).

To measure the gynoecium parameters, a line (green), indicating the position of the corresponding *x*-plane, was placed onto the cross-section of the ovary septum, and an orthogonal second line (red), indicating the corresponding *y*-plane, was placed along the dorsiventral plane of the gynoecium (Fig.2a). The gynoecium length and width (dorsiventral plane) were measured in the *y*-plane (Fig.2b). In the *x*-plane, the gynoecium width (septum) and length were measured (Fig.2c).

For measurement of the androecium parameters (anther and filament lengths), either the longitudinal images taken for the entire flower (Fig.2c) or, in older stages or when stamens were bent, longitudinal and transverse virtual sections of individual anthers were used. While measuring the filament length it was taken into account that the filament of *A. thaliana* is basifixed to the relatively short connective of the latrorse anthers. As far as possible, a discrimination between long and short stamens was made. The anther width is defined as the furthest distance from the two pairs of pollen sacs in cross-sectional view (Fig.2a).

**Fig 3:**
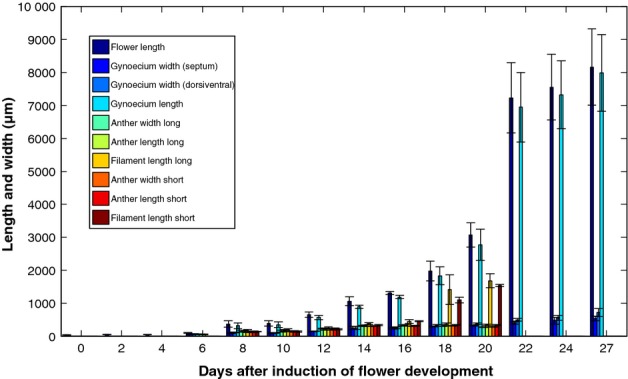
Morphometric data for *Arabidopsis thalianapAP1::AP1-GR ap1-1 cal-5* flower development and silique formation measured with micro computed tomography (micro-CT). For correspondence of harvesting days and developmental stages, replicate measurements and coefficients of variation, see Table 1.

All the data for the developmental parameters are summarized in Table 1 and visualized in a bar plot in Fig.3. On day 0, the primary floral apex was already present. Up to day 4, the continuously increasing flower length was the only parameter that could be measured because the individual flower organs were not yet differentiated. Floral organ initiation took place from day 2 onwards, when the sepal primordia appeared on the floral apex. From day 6 until day 20, all ten parameters were measured. Gynoecium width increased gradually, while gynoecium length grew exponentially until day 20 and nearly tripled between day 20 and day 22, followed by further gradual growth. The stamens were distinguishable from day 6 to day 20. The anthers tripled their width and length from day 6 to day 8. By contrast, the filaments developed and lengthened only slowly from day 8 onwards, followed by rapid, *c*. 3-fold growth just before anthesis on day 18. After anthesis, on day 22 the stamens abscised (see also Fig. S1G).

Of the 70 measured morphometric mean values listed in Table 1, the vast majority had a coefficient of variation below 15%. Almost all of them were below 25%, which lies within the normal range of biological variation (Table 1).

**Fig 4:**
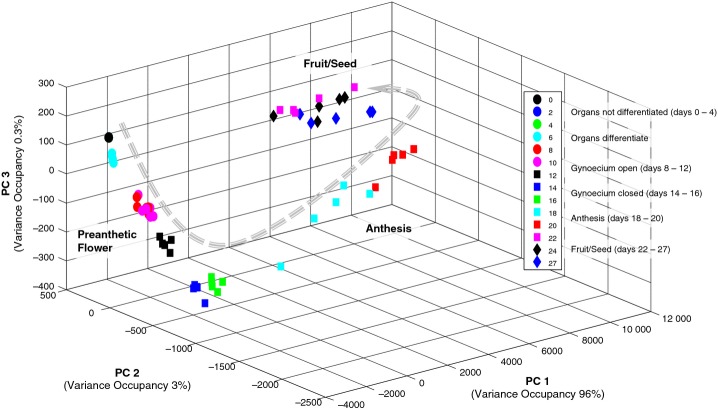
Principal components analysis (PCA) of morphometric micro computed tomography (micro-CT) data revealing the ontogenetric trajectory of *Arabidopsis thalianapAP1::AP1-GR ap1-1 cal-5* flower development from floral initiation to fruit development. Each dot represents a biological replicate from the harvesting day indicated in the key. For correspondence of harvesting day and developmental stage, see Table 1. The three phases of preanthetic flower, anthesis and fruit/seed development are distinguishable.

Floral development is a multivariate process defined by many different parameters. It is not possible to intuitively recognize the developmental trajectory derived from the multiple covariances of all parameters just by looking at the listed parameter table. Therefore, we applied multivariate statistical analysis which takes all parameters and their covariance or correlation into account for the explorative visualization of the developmental trajectory (Weckwerth, 2008). One of the main tools for this explorative analysis is principal components analysis (PCA). Using the morphometric parameters from Table 1 as variables, the different stages of flower development were visualized by PCA. PCA revealed the ontogenetic trajectory of flower and fruit development by plotting all replicate micro-CT analyses based on their covariance into a new 3D coordinate system spanned by the principal components PC1, PC2 and PC3 (Fig. 4, the grey arrow illustrates the developmental trajectory). For a better interpretation of the developmental trajectory, different phases of development were assigned to the PCA trajectory (three main phases: preanthetic flower, anthesis and fruit/seed; four subphases within the phase of preanthetic flower). The loadings of the PCA, which are a measure of the importance of variables for the visible trajectory (Weckwerth, 2008), can be found in Table S2. In the following, metabolite dynamics during development and the combined morphometric-metabolic trajectory are described.

### Metabolomic analysis of *A. thaliana* flower development

**Fig 5:**
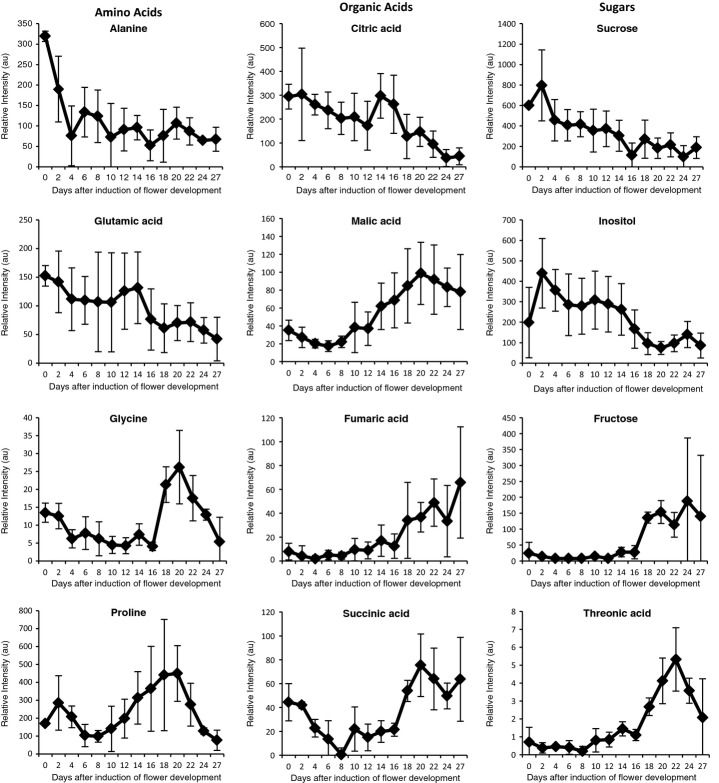
Metabolite profiles of *Arabidopsis thaliana pAP1::AP1-GR ap1-1 cal-5* flower samples. Samples were taken every 2 d over 27 d from induction of flower development by dexamethasone (day 0) to silique formation. Error bars show ± SD; *n* = 3–5 biological replicates. Shown are selected metabolites and their relative changes in concentration during flower development. For further details, see text. The full data set is available in Supporting Information Table S3. au, arbitrary units.

To compare the morphological data during flower development with metabolic changes, metabolites for all time-points from day 0 to day 27 were measured. More than 50 compounds could be identified and quantified by GC-MS (Table S3). The composition of sugars, organic and amino acids and other metabolites showed significant changes during flower and silique development (Fig.5). In the following, changes in the concentrations of selected metabolites are described. Concentrations of the sugar sucrose and the sugar alcohol inositol were relatively high during early development and then decreased, while at the same time fructose and glucose displayed low concentrations until day 16, and then a rapid increase soon after. The concentration of the sugar acid threonic acid was initially low but rose later in development, showing a peak at day 22. Analysis of the organic acids revealed that the concentration of citric acid was high early in development but decreased later. Malic acid, succinic acid and fumaric acid displayed constantly increasing concentrations over time. The amino acids alanine, glutamic acid and aspartic acid displayed high initial concentrations, but decreased thereafter. The glycine concentration displayed a peak during anthesis. The proline concentration was initially low but increased until day 20.

### Integration of metabolic and morphometric data during flower development – correlation of specific metabolites with their corresponding developmental stages

**Fig 6:**
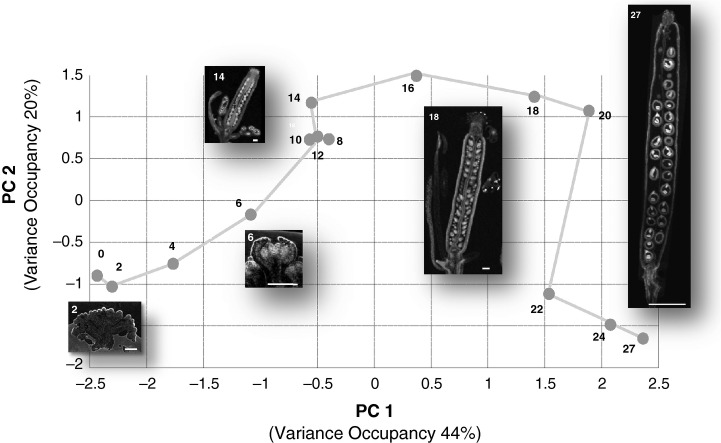
Principal components analysis of the combined metabolomic and morphometric data matrix of *Arabidopsis thaliana pAP1::AP1-GR ap1-1 cal-5*. Mean values for metabolite data as well as morphometric data from micro computed tomography (micro-CT) measurements were transformed, aligned and combined into one data matrix using covain (Sun & Weckwerth, 2012). This integrative data matrix was analysed by principal components analysis (PCA) using covain. PCA revealed the integrative molecular and morphometric ontogenetic trajectory of *A. thaliana* flower development and silique formation, shown as a grey line. Every point on this trajectory represents the combined and transformed vector of metabolite and morphometric mean values of one developmental stage and is labelled with the corresponding day. For correspondence of harvesting day and developmental stage, see Table 1. To exemplify the different stages of development, pictures of micro-CT measurements have been added. Bars: days 2–18, 100 μm; day 27, 1000 μm.

**Fig 7:**
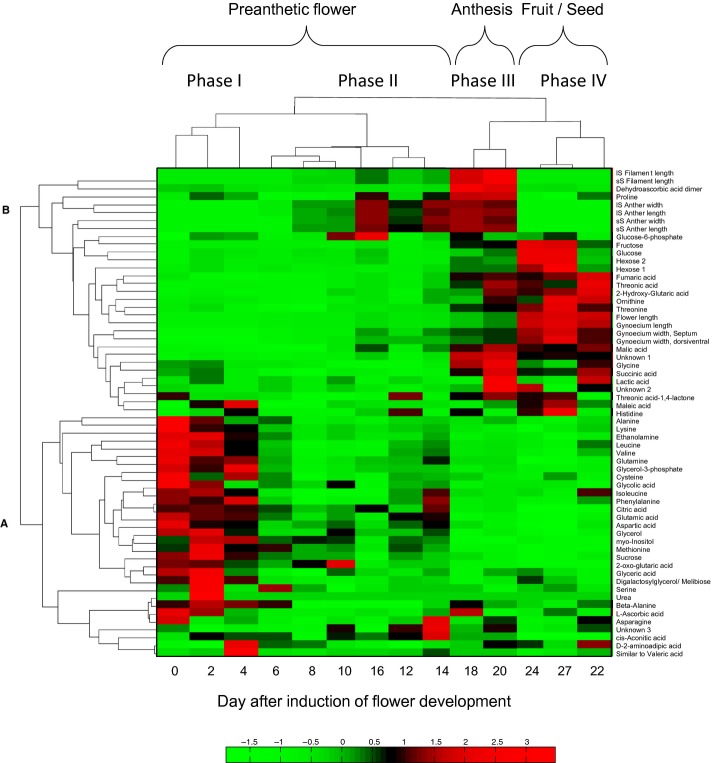
Integrative bi-clustering analysis of variables (metabolites and morphometric parameters) versus samples (harvesting time-points in days) corresponding to specific developmental flower stages of *Arabidopsis thaliana pAP1::AP1-GR ap1-1 cal-5* (see Table 1). The developmental stages are clustered into the three major clusters and four subphases as discussed in the text. In the vertical cluster A, early developmental stages are associated with high concentrations of certain metabolites, such as sucrose, glutamine and asparagine. In cluster B, late development is associated with higher concentrations of a different set of metabolites, including proline, glucose and fructose (for more details see the Discussion section). Bi-clustering was performed with covain (Sun & Weckwerth, 2012) and uses average linkage of Euclidean distance between groups as the metric. The colour scale is based on standardized values.

The mean values of the biological replicates for each developmental stage were used to integrate metabolite and morphometric data on flower development. Each data set was uploaded and normalized using the range-scaling function implemented in covain (Sun & Weckwerth, 2012) to allow the comparison of metabolite and morphometric data. Data alignment of the two data sets was then performed using an alignment function in covain (Sun & Weckwerth, 2012), resulting in an integrative data matrix comprising normalized metabolite concentrations and morphometric parameters of flower development. The data were analysed using the PCA function of covain (Fig.6). Based on the covariance of morphometric and metabolic parameters, the ontogenetic trajectory of flower development becomes visible by plotting PC1 and PC2. The association of morphometric and metabolite data is revealed by the loadings of PC1 and PC2 (Weckwerth, 2008). In Fig. S2 and Table S2 the loadings are shown. Gynoecium and anther parameters had a strong influence on the trajectory, as well as the sugars fructose, glucose, sucrose and myo-inositol, the amino acids glutamic acid and glutamine, and the organic acids malate and citrate. The morphometric and metabolite parameters were further analysed by bi-clustering analysis using covain (Fig.7). Two major clusters were observed: all metabolites and morphometric parameters taken from the preanthetic flower samples (days 0–16) grouped together and were further separated into three subclusters (days 0–2, day 4 and days 6–16); the other cluster was split into two subgroups comprising days 18–20, centred on anthesis, and days 22–27, during fruit/seed development. Altogether four phases could be observed (phase I: days 0–4; phase II: days 6–16; phase III: days18–20, and phase IV: days 22–27). On the basis of this clustering analysis, metabolites that were significantly associated with different developmental stages were identified. Phase I was associated with the amino acids alanine, glutamine, valine and aspartate as well as organic acids such as citrate and components of lipid and membrane metabolism, namely glycerol-3-phosphate, glycolate and ethanolamine. At the same time, hexoses stayed at relatively low concentrations. Transitional medium concentrations of inositol and sucrose connected phase I with phase II before they decreased significantly, while glucose and fructose concentrations increased. On the morphometric side, phase II was characterized by the growth of the anthers and subsequently, at day 14, by an increase of gynoecium width and length. The proline concentration increased from day 14 to day 20, thereby bridging phases II and III, and peaked at day 20, before decreasing again. Days 14 and 16 marked the transition to phase III and were characterized by pronounced morphometric changes (growth of anthers) and slightly increased concentrations of glucose-6-phosphate. Another metabolite associated with this development was malate, whose concentration slightly increased up to day 16 and peaked at day 20. Day 22 bridged anthesis and fruit development and was characterized by higher concentrations of the organic acids fumarate, alpha-hydroxy-glutarate and threonate. Phase IV corresponded to fruit development and showed high concentrations of hexoses. Concomitantly, the gynoecium enlarged rapidly.

## Discussion

### Morphometric analysis

Micro-CT permits better visualization of the morphological processes in flower development than has previously been achieved. Not only does it produce 3D images, but, via virtual slicing, it also allows one to focus on exactly the plane or section of interest. Importantly, as a consequence of its nondestructive nature, this method is also extremely flexible, because plane selection is reversible at any time. This saves a lot of time compared with analysis via LM, which not only requires time-consuming preparation and analysis procedures but is also limited by the irreversibility of section planes. Yet, LM images of microtome-sectioned and specifically stained material deliver important additional qualitative information, such as the presence of certain plant substances and tissue activity, which cannot be detected via micro-CT (see the red-purple staining of mucilage in pollen tube transmitting tissue at day 18, and dark-blue meristematic cells in all developmental stages shown in Fig.1). LM is important to evaluate stages of flower development with higher resolution (e.g. tetrads; see Fig. S1A). For investigation of the flower surface, SEM still provides the highest resolution. However, SEM reduces a 3D object to a 2D image, leading to inaccuracy of organ size measurements, especially when dome-shaped inflorescences and curved organs are investigated. Also, overlapping structures cannot be accurately measured. Micro-CT is therefore currently the best tool for obtaining reliable and accurate morphometric data on outer- as well as inner-organic measurements.

Based on the morphometric micro-CT data for 10 characteristic developmental parameters (Table 1), a very pronounced ontogenetic trajectory of flower development can be observed in Fig.4. First of all, flower development can be subdivided into three phases: preanthetic development, anthesis, and fruit/seed development. Within these three main phases, further subphases can be distinguished. The first subphase includes days 0 to 4 and comprises very early development of the primary floral meristem, closely followed by a second subphase, in which, on day 6, the floral organs are still very small but clearly recognizable. Another subphase comprises days 8–12. During this subphase, the gynoecium forms a distally open tube and ovule formation and pollen development are under way. Days 14–16 are characterized by a closed gynoecium and the development of stigmatic papillae.

Days 18–20 mark a transition phase in the PCA (Fig. 4). They can be identified as the time of anthesis and the initiation of seed/fruit development. Anther dehiscence and the presence of pollen on the stigma can be observed (Figs4, S1D,E).

Days 22–27 are grouped together on the PCA and represent differentiation and growth of seeds and siliques. Although not distinguishable on PC1, the three days can be separated by PC3. PC1 is dominated by flower length and gynoecium length. PC2 is dominated by filament length and PC3 by anther width, anther length, gynoecium width (septum), gynoecium width (dorsiventral) and flower length (see Table S2 with PC loadings).

As the progress of development and plant growth are strongly influenced by environmental parameters, it is more useful to compare plants of a certain developmental stage rather than plants of similar chronological age (Boyes *et al.*, 2001). Thus, the stages of flower development of an *A. thaliana* wild-type plant described by Smyth *et al*. (1990) were assigned to days of sampling of our mutant after initiation of flower development in this study (Table 1). The attribution showed clearly that, during certain periods in floral ontogeny, several developmental stages are passed through rapidly. For example, stages 4, 5 and 6 were assigned to day 6 and stages 7 and 8 were assigned to day 8 (see Table 1). This accelerated developmental process correlates with the differentiation of the floral organs. By contrast, there are also periods with extended developmental stages, for example, fruit development (days 24–27). During this phase the fruit is already differentiated and development is dominated by growth.

### The combined metabolomic and morphometric analysis reveals distinct mechanisms of metabolic adjustment during flower morphogenesis

Flower initiation in *A. thaliana* wild-type plants takes place continuously in an infinite spiral. Thus, the stem holds flowers of many developmental stages. To be able to measure the different developmental flower stages separately and in order to obtain enough homogenous flower material for the metabolomic analysis, we used the *pAP1::AP1-GR ap1-1 cal-5* mutant of *A. thaliana*. This mutant has two important characteristics which were most suitable for our purpose. First, flower development can be induced by application of the synthetic hormone dexametasone and consequently all flowers of one inflorescence develop synchronously in the early stages. Therefore, the date of harvest correlates with the stage of flower development. This allowed us to measure definite developmental stages. Secondly, the cauliflower habitus expressed by this mutant provides an increased number of flowers on an individual stem and thus more sample material, which is needed especially for the metabolic analysis of the small preanthetic stages.

The metabolic profile obtained by mass spectrometry showed clearly that the metabolism changed in unison with all phases of flower development. There was a pronounced dynamic of metabolism throughout flower development. These data, however, need to be carefully discussed. In particular, the concerted initiation of many flowers at the same time might have an influence on the source–sink distribution of the metabolites. In the following, the observed dynamics of specific compound classes are discussed.

**Fig 8:**
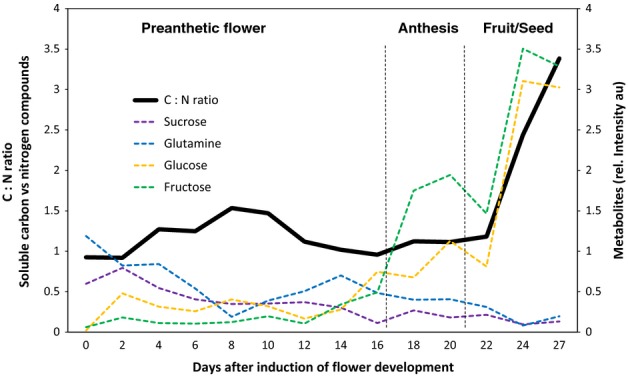
Carbon (C) : nitrogen (N) ratio of readily available soluble metabolites during *Arabidopsis thaliana pAP1::AP1-GR ap1-1 cal-5* flower development and silique formation. For correspondence of harvesting day and developmental stage, see Table 1. For further details, see the Discussion section. au, arbitrary units.

Sugars have important regulatory functions in controlling metabolism, stress resistance, growth and development in plants (Smeekens *et al.*, 2010). In photosynthetic, sugar-producing and sessile organisms, maintenance of energy homeostasis requires mechanisms to account for the physiological and developmental plasticity observed in plants. In recent years, the crucial role of sugars as signalling molecules and their dramatic effects on plant growth and development have been described (Koch, 2004; Rolland *et al.*, 2006). During flower development, assimilates are transported from the leaf to the floral meristem. Sucrose and inositol as well as other carbohydrates such as melibiose accumulate during the very early stages of floral development and decrease in the later stages (Figs7). Sugars are consumed for ATP production, and channelled into membrane and cell wall synthesis and other biosynthetic pathways necessary to build up the floral structures. In addition to their role as catabolic and anabolic compounds, a metabolic sensing mechanism as well as the control of C : N ratios can be assumed to play an important role in the developing plant tissue. Initiation of flower development is accompanied by a high C : N ratio in the phloem sap (Corbesier *et al.*, 2002). The C : N ratio had previously only been measured in leaves or phloem sap and not directly in flower tissue from very early stages of development. Fig.8 shows the C : N ratio of readily available soluble metabolic compounds (sugars, organic acids and others versus amino acids) over the course of floral development. Here, the C : N ratio is characterized not only by initially high sucrose concentrations but also by high concentrations of the amino acids alanine, glutamine, glutamate and aspartate in the early stages of flower development. These amino acids are all involved in transamination processes and N assimilation. Therefore, we hypothesize that, in the early stages of floral induction, there is high N assimilation activity in the developing tissue. Genes involved in N assimilation processes (At5g37600 and At1g66200; glutamine synthetases and At5g07440; glutamate dehydrogenase) are highly up-regulated in these early stages; they are depicted as a cluster of highly expressed genes in the very early stages of flower development in the study of Wellmer *et al*. (2006). These increased N assimilation processes may lead to increased concentrations of other amino acids such as lysine, leucine, valine, cysteine, methionine and phenylalanine in the very early stages of floral induction. Free amino acids in the floral meristem allow high translational activity and *de novo* synthesis of proteins. At the same time, high metabolic activity of respiratory energy metabolism is indicated by the stage-specific dynamics of the organic acids citrate, malate and fumarate.

In the later stages of flower development during silique and seed development, the C : N ratio of ready available soluble metabolites increases significantly as a result of a rapid increase in glucose and fructose concentrations (Fig.8). Amino acids decline and are putatively stored in storage proteins.

### A proposed model for the adjustment of C : N partitioning during flower morphogenesis

It is very likely that transcriptional regulation plays an important underlying role in establishing the metabolic composition in developing flowers. Therefore, transcription factors, which are highly expressed in early flower stages, are interesting candidates for involvement in the upstream regulatory processes determining the C : N ratio. The C : N balance in plants is not only controlled by the genes of N assimilation such as glutamine synthetase and glutamate dehydrogenase, but it is also regulated by several members of a large group of transcription factors, the basic leucine zipper (bZIP) gene family involved in sugar-sensing processes (Hanson *et al.*, 2008; Smeekens *et al.*, 2010; Ma *et al.*, 2011). Among the genes that were found to be highly expressed in the early phase of floral initiation in the study of Wellmer *et al*. were several bZIP transcription factors (At4g35900 (bZIP 14), At2g17770 (bZIP27) (Abe *et al.*, 2005) and especially At4g34590 (bZIP11) (Wellmer *et al.*, 2006)). Remarkably, the metabolite profiles of the early developmental stages observed in our study correspond to metabolic changes observed in stable bZIP11 over-expressor lines and plant lines with transiently increased expression of bZIP11. In these studies proline concentrations were found to be low, and phenylalanine and asparagine concentrations were high compared with wild-type (Hanson *et al.*, 2008). In Fig.7 our metabolite data reveal a similar pattern in the two vertical clusters A and B. Cluster A represents the metabolite associations with early development (higher bZIP11 expression rates), including high concentrations of sucrose, glutamine, glutamic acid, asparagine and phenylalanine. Conversely, cluster B reveals metabolite associations with late development (lower bZIP11 expression rates) including high concentrations of proline, glucose, fructose and others. Therefore, the separation into two main metabolic phases, as well as the shift in the C : N ratio, could be the result of a metabolic switch, regulated at least partially by bZIP transcription factors including bZIP11 and sugar-sensing mechanisms. The high levels of bZIP11 in the earlier stages of flower development, and its decrease in expression in later stages, could contribute considerably to the observed changes in metabolite concentrations. However, as flower development is a multifactorial process, bZIP transcription factors probably comprise only one part of a complex interwoven regulatory network. Hence, further investigation is necessary to elaborate the potential role of bZIPs in flower development.

The integration of metabolite profiles with high-resolution morphometric data allowed a very detailed view of metabolic reprogramming during flower development and opened up novel interrelationships between metabolism and specific stages of flower development. Genetic control and metabolic feedback are relevant mechanisms during flower development and can be described by an integrative ontogenetic trajectory as shown in this study for *A. thaliana*. Future studies will focus on refining correlations between metabolomic profiles and developmental stages and will also be carried out in other angiosperm lineages, integrating molecular and morphological ontogenetic trajectories.

## Supporting Information

Fig S1 Different phases during *Arabidopsis thaliana pAP1::AP1-GR ap1-1 cal-5* flower development. **Fig. S2** PCA loadings of the combined morphometric and metabolic covariance matrix of Fig. 6.

Table S1 Micro-CT scanning conditions

Table S2 PCA loadings of PCAs in 4 and 6

Table S3 Metabolite profiles of *Arabidopsis thaliana pAP1::AP1-GR ap1-1 cal-5* from induction of flower development to silique formation
