# Supplementary material for: Metabolism and development – integration of micro computed tomography data and metabolite profiling reveals metabolic reprogramming from floral initiation to silique development
Source: New Phytol. 2013 Dec 18;202(1):322–35. doi: 10.1111/nph.12631 (PMC4283998; doi:10.1111/nph.12631)
Supplement: Fig S1 — Different phases during Arabidopsis thaliana pAP1::AP1-GR ap1-1 cal-5 flower development. Fig. S2 PCA loadings of the combined morphometric and metabolic covariance matrix of Fig. 6. [file nph0202-0322-sd1.pptx]

## Slide 1
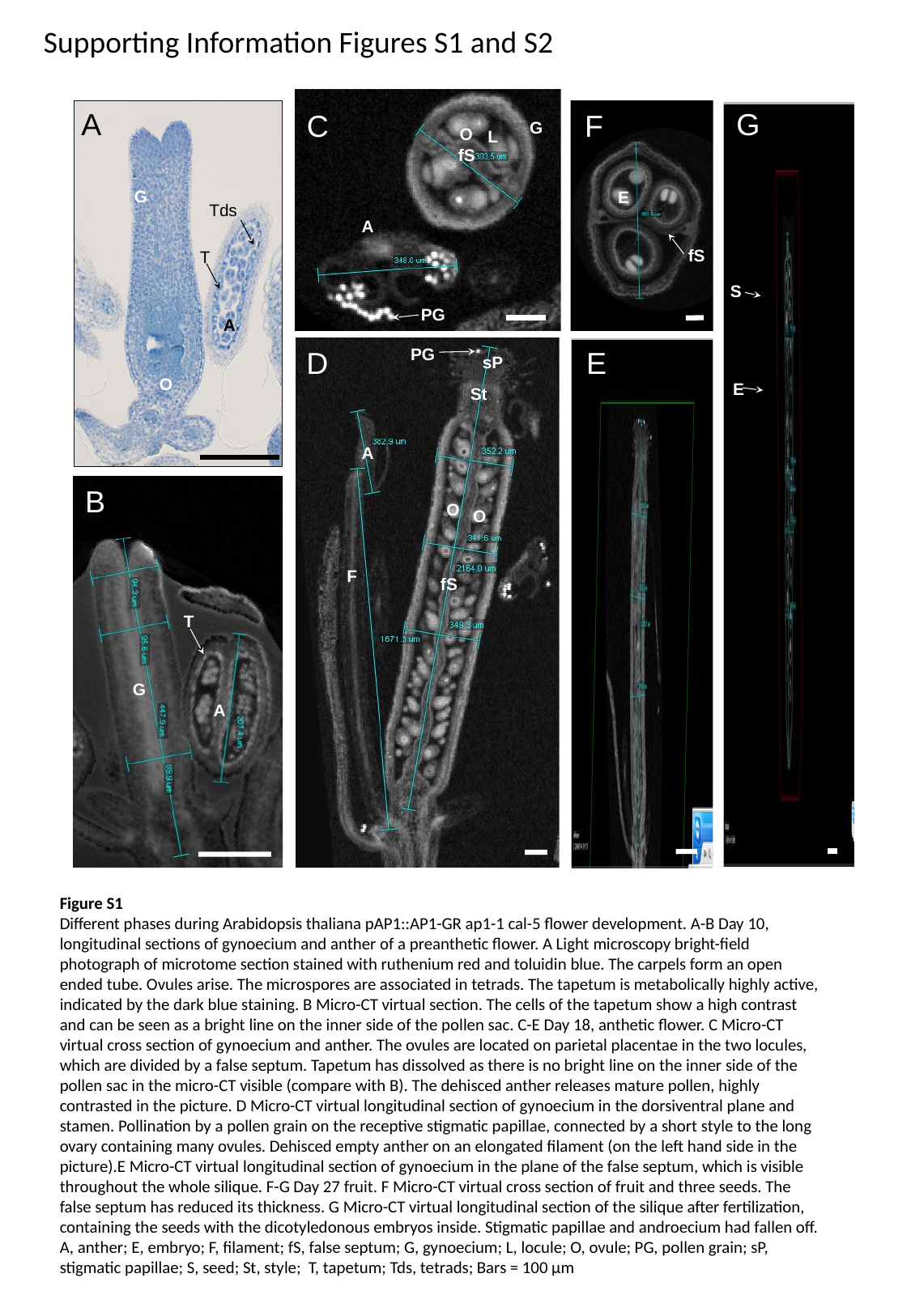

Supporting Information Figures S1 and S2
A
G
C
C
F
F
G
G
O
L
fS
G
E
Tds
A
fS
T
S
PG
A
D
D
E
E
PG
sP
O
E
St
A
B
B
O
O
F
fS
T
A
G
Figure S1
Different phases during Arabidopsis thaliana pAP1::AP1-GR ap1-1 cal-5 flower development. A-B Day 10, longitudinal sections of gynoecium and anther of a preanthetic flower. A Light microscopy bright-field photograph of microtome section stained with ruthenium red and toluidin blue. The carpels form an open ended tube. Ovules arise. The microspores are associated in tetrads. The tapetum is metabolically highly active, indicated by the dark blue staining. B Micro-CT virtual section. The cells of the tapetum show a high contrast and can be seen as a bright line on the inner side of the pollen sac. C-E Day 18, anthetic flower. C Micro-CT virtual cross section of gynoecium and anther. The ovules are located on parietal placentae in the two locules, which are divided by a false septum. Tapetum has dissolved as there is no bright line on the inner side of the pollen sac in the micro-CT visible (compare with B). The dehisced anther releases mature pollen, highly contrasted in the picture. D Micro-CT virtual longitudinal section of gynoecium in the dorsiventral plane and stamen. Pollination by a pollen grain on the receptive stigmatic papillae, connected by a short style to the long ovary containing many ovules. Dehisced empty anther on an elongated filament (on the left hand side in the picture).E Micro-CT virtual longitudinal section of gynoecium in the plane of the false septum, which is visible throughout the whole silique. F-G Day 27 fruit. F Micro-CT virtual cross section of fruit and three seeds. The false septum has reduced its thickness. G Micro-CT virtual longitudinal section of the silique after fertilization, containing the seeds with the dicotyledonous embryos inside. Stigmatic papillae and androecium had fallen off. A, anther; E, embryo; F, filament; fS, false septum; G, gynoecium; L, locule; O, ovule; PG, pollen grain; sP, stigmatic papillae; S, seed; St, style; T, tapetum; Tds, tetrads; Bars = 100 µm

## Slide 2
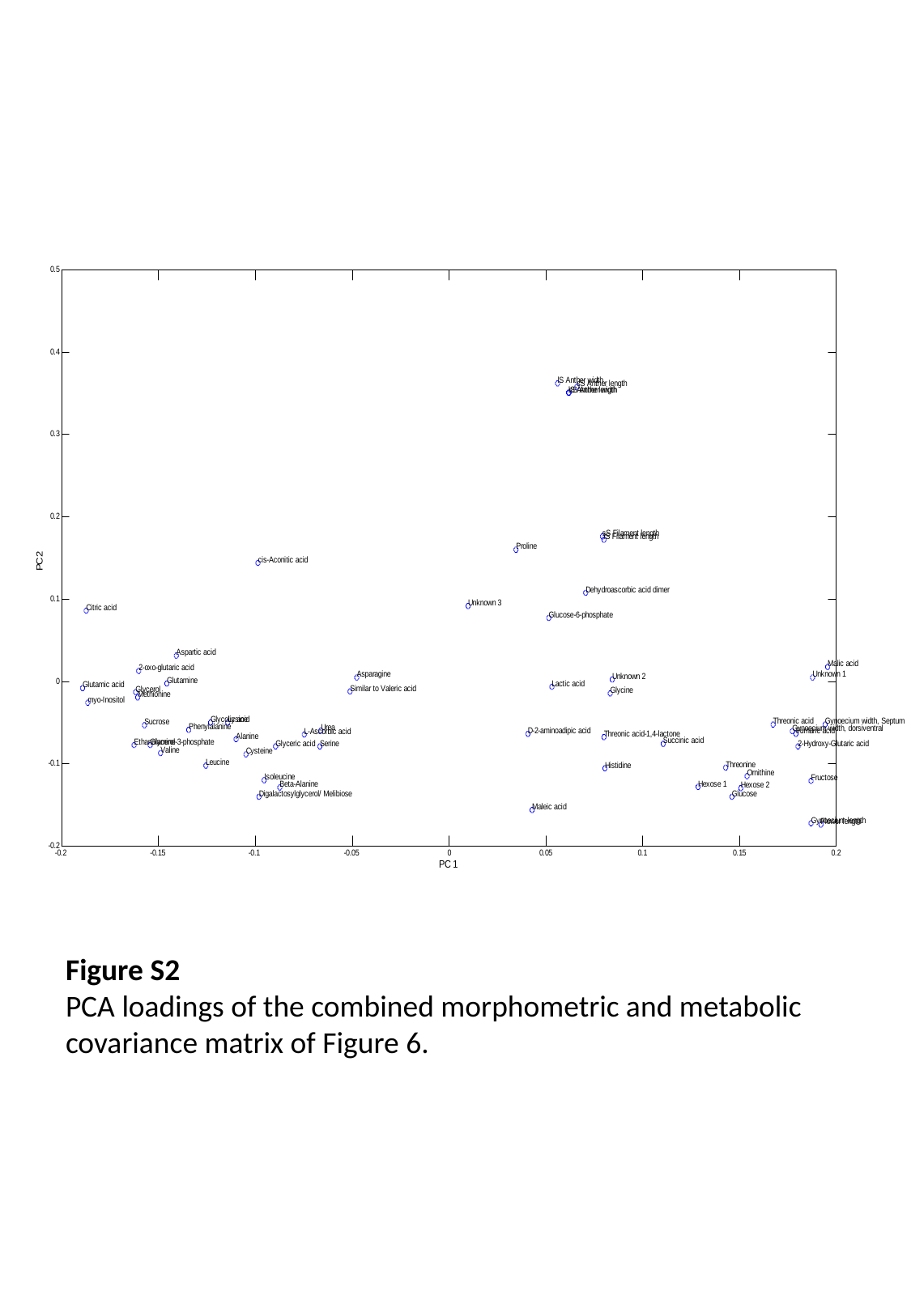

Figure S2
PCA loadings of the combined morphometric and metabolic covariance matrix of Figure 6.
