# Supplementary material for: Metabolism and development – integration of micro computed tomography data and metabolite profiling reveals metabolic reprogramming from floral initiation to silique development
Source: New Phytol. 2013 Dec 18;202(1):322–35. doi: 10.1111/nph.12631 (PMC4283998; doi:10.1111/nph.12631)
Supplement: Table S1 — Micro-CT scanning conditions [file nph0202-0322-sd2.docx]

**Supporting Information Table S1 micro-CT scanning conditions**

| **Day after initiation of flower development** | **Stage according to Smyth *et al.* (1990)** | **Critical point drying and sputter-coating with gold** | **Acceleration voltage [kV]** | **Source current [µA]** | **Exposure time [s]** | **Pictures per sample** | **Camera binning** | **Optical magnification** | **Pixel size [µm]** |
| --- | --- | --- | --- | --- | --- | --- | --- | --- | --- |
|  |  |  |  |  |  |  |  |  |  |
| **0** | **1** | **+** | **40** | **100** | **20** | **1200** | **1** | **20** | **0,4** |
|  |  |  |  |  |  |  |  |  |  |
| **2** | **2** | **+** | **60** | **100** | **40** | **1200** | **1** | **20** | **0,4** |
|  |  |  |  |  |  |  |  |  |  |
| **4** | **3** | **+** | **35** | **84** | **60** | **728** | **1** | **20** | **0,4** |
|  |  |  |  |  |  |  |  |  |  |
| **6** | **4; 5; 6** | **+** | **40** | **100** | **20** | **1200** | **1** | **20** | **0,5** |
|  |  |  |  |  |  |  |  |  |  |
| **8** | **7; 8** | **+** | **40** | **100** | **30** | **1600** | **1** | **10** | **0,7** |
|  |  |  |  |  |  |  |  |  |  |
| **10** | **9** | **+** | **40** | **100** | **20** | **1600** | **1** | **10** | **0,8** |
|  |  |  |  |  |  |  |  |  |  |
| **12** | **10** | **+** | **40** | **62** | **20** | **1200** | **1** | **10** | **1,0** |
|  |  |  |  |  |  |  |  |  |  |
| **14** | **11** | **-** | **40** | **100** | **5** | **728** | **2** | **10** | **1,8** |
|  |  |  |  |  |  |  |  |  |  |
| **16** | **12** | **-** | **40** | **100** | **10** | **728** | **2** | **10** | **1,8** |
|  |  |  |  |  |  |  |  |  |  |
| **18** | **13** | **-** | **40** | **100** | **5** | **728** | **2** | **10** | **2,0** |
|  |  |  |  |  |  |  |  |  |  |
| **20** | **15** | **-** | **40** | **100** | **5** | **728** | **2** | **10** | **1,8** |
|  |  |  |  |  |  |  |  |  |  |
| **22** | **16** | **-** | **40** | **100** | **5** | **728** | **2** | **10** | **1,8** |
|  |  |  |  |  |  |  |  |  |  |
| **24** | **17** | **-** | **40** | **100** | **10** | **728** | **1** | **4** | **2,1** |
|  |  |  |  |  |  |  |  |  |  |
| **27** | **17** | **-** | **40** | **100** | **5** | **728** | **2** | **10** | **1,8** |
|  |  |  |  |  |  |  |  |  |  |
